# Exposomic determinants of immune-mediated diseases: Special focus on type 1 diabetes, celiac disease, asthma, and allergies: The HEDIMED project approach

**DOI:** 10.1097/EE9.0000000000000212

**Published:** 2022-06-08

**Authors:** Jutta E. Laiho, Olli H. Laitinen, Johannes Malkamäki, Leena Puustinen, Aki Sinkkonen, Juha Pärkkä, Heikki Hyöty

**Affiliations:** a Faculty of Medicine and Health Technology, Tampere University, Tampere, Finland; b Natural Resources Institute Finland, Turku, Finland; c VTT Technical Research Centre of Finland Ltd, Tampere, Finland; d Fimlab Laboratories, Pirkanmaa Hospital District, Tampere, Finland

**Keywords:** Allergy, Asthma, Celiac disease, Diet, Interventions, Microbiome, OMICS, Satellite data, Toxicants, Type 1 diabetes

## Abstract

The incidence of immune-mediated diseases (IMDs) is increasing rapidly in the developed countries constituting a huge medical, economic, and societal challenge. The exposome plays an important role since genetic factors cannot explain such a rapid change. In the Human Exposomic Determinants of Immune Mediated Diseases (HEDIMED) project, altogether 22 academic and industrial partners join their multidisciplinary forces to identify exposomic determinants that are driving the IMD epidemic. The project is based on a combination of data and biological samples from large clinical cohorts constituting about 350,000 pregnant women, 30,000 children prospectively followed from birth, and 7,000 children from cross-sectional studies. HEDIMED focuses on common chronic IMDs that cause a significant disease burden, including type 1 diabetes, celiac disease, allergy, and asthma. Exposomic disease determinants and the underlying biological pathways will be identified by an exploratory approach using advanced omics and multiplex technologies combined with cutting-edge data mining technologies. Emphasis is put on fetal and childhood exposome since the IMD disease processes start early. Inclusion of several IMDs makes it possible to identify common exposomic determinants for the diseases, thus facilitating the development of widely operating preventive and curative treatments. HEDIMED includes data and samples from birth cohorts and clinical trials that have used exposomic interventions and cell and organ culture models to identify mechanisms of the observed associations. Importantly, HEDIMED generates a toolbox that offers science-based functional tools for key stakeholders to control the IMD epidemic. Altogether, HEDIMED aims at innovations, which become widely exploited in diagnostic, therapeutic, preventive, and health economic approaches.

**What this study adds**

In this review, we describe the design of the HEDIMED project, which participates in the common European Union (EU) effort to broaden the knowledge about the exposome and its effect on human health. HEDIMED is the largest study so far to tackle the concept of the exposome and its relation to the development of immune-mediated diseases. HEDIMED will be taking into account the complex interplay between external and internal exposomes and the key molecules and pathways operating in early life to identify disease mechanisms and risk and protective exposures that are critical for the development of T1D, CD, allergy, and asthma.

## Introduction

**Figure 1.**
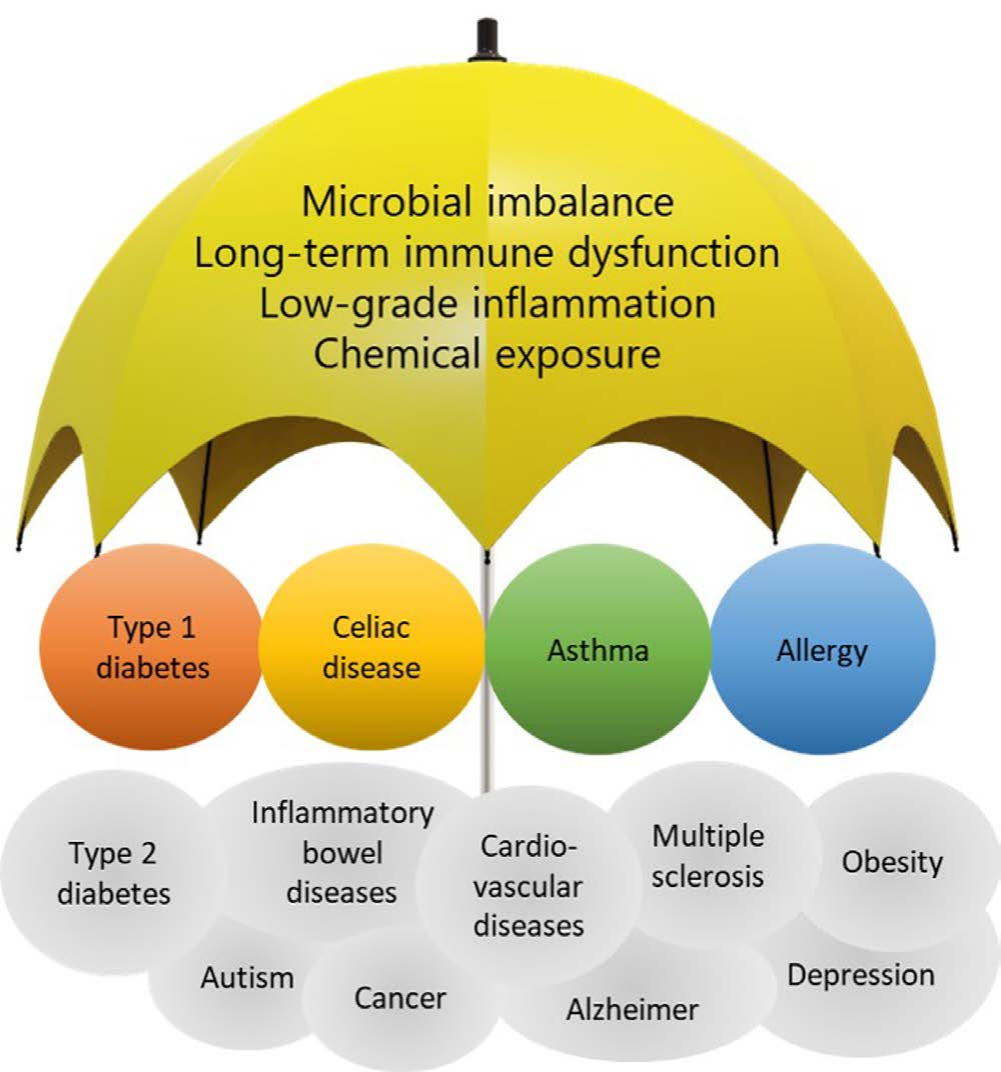
Many noncommunicable diseases have been suggested to share underlying risk factors. These factors include microbial imbalance and associated low-grade inflammation, exposures to environmental toxicants and infections, loss of environmental microbial biodiversity, and changes of dietary habits, which together can disturb the balance of immune homeostasis disposing individuals to IMDs. These risk factors marked in the parasol cast a shadow over many IMDs and are thought to be at least partially the mediators in the pathogenesis of the IMDs relevant for HEDIMED as well (type 1 diabetes, celiac disease, asthma, and allergies). Figure adapted from Haahtela et al.^7^

During the past decades, the incidence of many chronic conditions, such as respiratory, allergic, autoimmune, metabolic, and mental diseases, has increased in developed countries, presumably due to urbanization-associated changes in our lifestyle, exposure to environmental biodiversity^1^ and chemical environment. The rapidly changing environment and lifestyles have in a way outran the human immune system, as the immune system has not had enough time to adapt to the change. This may manifest in several ways, including microbial imbalance (gut dysbiosis), long-term immune dysfunction, and low-grade inflammation, which may turn to risk factors for several diseases. The increasing chemical exposure in the form of various toxicants and pollutants including, for example, polyaromatic hydrocarbons, microplastics, and endocrine disruptors can also have harmful effects on the immunological and endocrinological homeostasis either directly or via. their effects on the environmental or human microbiome.^2–5^ Not surprisingly, many of these risk factors have been suggested to be shared between several noncommunicable diseases, depicted in Figure 1.^6,7^

Included within these noncommunicable diseases are immune-mediated diseases (IMDs), whose rapid increase constitute a huge medical, economic, and societal challenge.^8^ IMDs are often life-long diseases with significant reduction in the quality and even the length of life. Their pathogenesis lies in the loss of immunological tolerance to antigens present in body’s own cells (autoantigens) or to otherwise harmless environmental antigens (e.g., allergens, commensal bacteria) leading to inflammation and cell damage.^9–11^ Among the most common IMDs are allergic diseases, asthma, type 1 diabetes (T1D), and celiac disease (CD).^12^

There is no curative treatment for IMDs apart from some allergies, where allergen desensitization treatment can lead to the loss of symptoms.^13,14^ Other available treatments can alleviate symptoms but they cannot cure the disease or prevent completely long-term complications—moreover, there are significant costs and side effects of modern IMD therapies. Therefore, there is an unmet need to prevent IMDs or alleviate their symptoms with better approaches.

Development of preventive interventions requires profound understanding of the disease mechanisms, including key molecules and pathways. Based on the current consensus, these mechanisms operate already at early life including the in utero period,^15,16^ when the first subclinical signs of IMDs often appear^17^ and when the immune system is under rapid transition from an immature stage to the mature adult stage. As a significant component in these processes, the crosstalk between the commensal microflora^18,19^ and the child’s maturating immune system has received special attention in the field.^20^ Thus, these early events are the most promising targets for preventive interventions.

**Figure 2.**
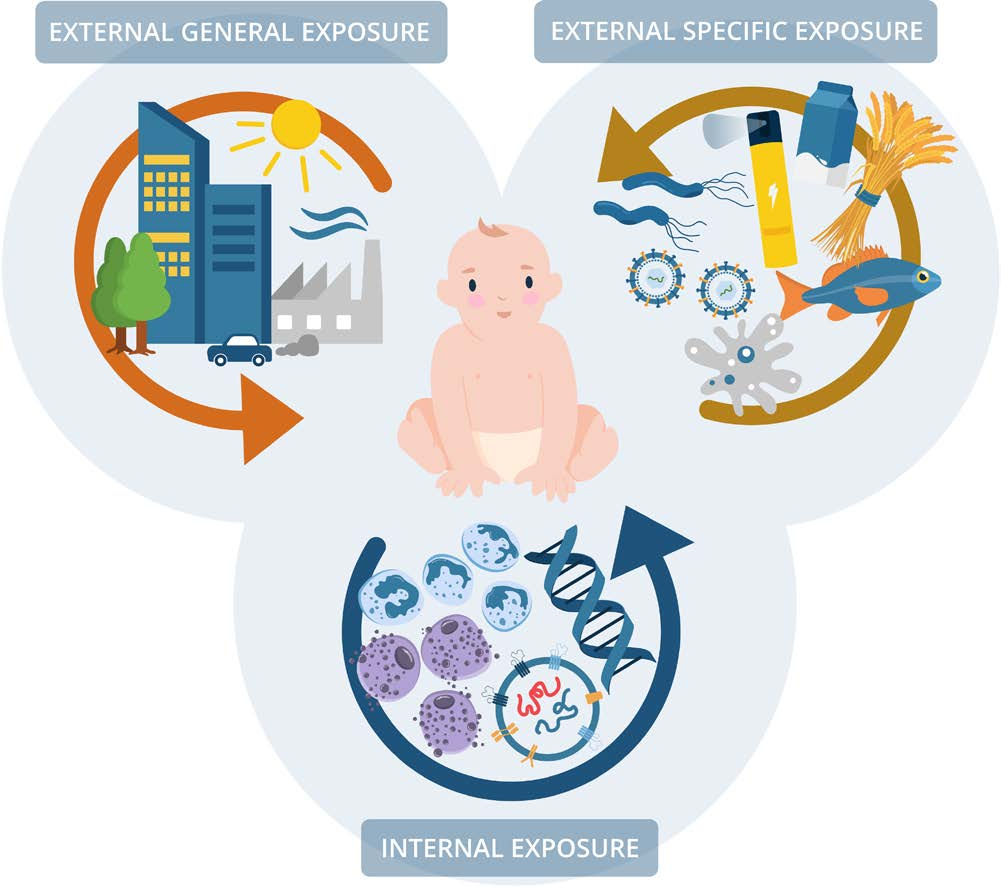
The three categories of the exposome. General and specific external exposome and internal exposome interact closely with each other and influence the rapidly evolving immune system at early life, and these exposomic factors can either protect or dispose to IMDs.

Although the risk of IMDs is modulated by genetic factors, they cannot explain their rapidly increasing incidence, and therefore the environment, or exposome, must play an important role.^21^ The term exposome refers to the environmental, that is, nongenetic, drivers of disease that influence the individual from conception onwards.^22^ The exposome is generally divided into three categories that interact with each other. The general external exposome covers socioeconomic and climatic factors and the living environment, whereas the specific external exposome covers factors like pollutants, infections, and lifestyle. The internal exposome, on the other hand, includes factors within the body such as the microflora, inflammation, metabolism, and hormones (Figure 2).

Early life exposome modulates the phenotype by influencing the developing homeostatic systems and pathways. These modulating exposomic determinants can include both disease promoting and preventing factors.^23–27^ According to the prevailing hypothesis, the balance between these exposures, together with the variation in host’s response to these exposures partially regulated by genetics and epigenetics, eventually determines whether the disease-causing process initiates.

Human Exposomic Determinants of Immune Mediated Diseases (HEDIMED) focuses on these early life events that occur during prenatal and postnatal life when both the internal and external exposome affect the development of the immune system and the initiation and progression of T1D, CD, allergy, and asthma—all known to be associated with specific genetics but having had a rising incidence during the past decades. T1D is a heterogeneous disease, characterized by selective destruction of insulin-producing pancreatic beta cells by a yet unknown mechanism, leading to loss of insulin production and requiring a life-long daily administration of insulin. The primary cause to the onset of CD is gluten found in rye, barley, and wheat, causing an autoimmune reaction against small-bowel mucosa, but also exposomic factors such as infections can have an influence on triggering CD. CD and T1D also share common genetic components, whose influence can be modulated by exposomic factors. The only available treatment for CD is gluten-free diet. Allergy is immune system’s abnormal response to harmless substances, and it can be manifested in different organs including nasal mucosa, sinuses, lower airways, skin, gastrointestinal tract, and eyes. Exposomic factors such as infections, pollutants, and changes in diet have been suspected to partially explain the current epidemic. Asthma is a chronic condition with swelling in the small airways in the lungs, which is caused by an overactive immune system. Certain volatile compounds and other toxicants, and different allergens are among the exposomic factors that can trigger the asthma and, for example, rhinoviruses cause asthma exacerbations. The mechanisms leading to these diseases are believed to have partly common origins with several other IMDs. Supporting this is a study where populations with similar genetic background were compared in Finland and Russian Karelia. As a result, the incidence of both allergic diseases and autoimmunity disorders, T1D, CD, and thyroid autoimmunity, were considerably lower in Russian Karelia.^28^ It is noteworthy that welfare and hygiene differences were radical between these two regions, with remarkably higher pathogen load in Russian Karelia.

Here, we describe the design of the HEDIMED project, which participates in the common EU effort to broaden the knowledge about the exposome and its effect on human health. HEDIMED is the largest study so far to tackle the concept of the exposome and its relation to the development of immune-mediated diseases. HEDIMED will be taking into account the complex interplay between external and internal exposomes and the key molecules and pathways operating in early life to identify disease mechanisms and risk and protective exposures that are critical for the development of T1D, CD, allergy, and asthma.

## Study hypothesis

HEDIMED project is based on the hypothesis that exposomic factors can markedly modulate the risk of IMDs, particularly when occurring during the first years of life, including the fetal period, when the immune system maturates rapidly and when the disease-predisposing responses often develop.^29–32^ These risk modulating factors can either increase or decrease the risk of IMDs. Risk and protective factors can overlap, for example, some infections may increase the risk by causing tissue damage in target organs, while other infections may decrease the risk by activating immunoregulatory pathways. In addition, the effect of these factors can be influenced by several modifying factors. For example, the effect of dietary gluten on the development of celiac disease is modified by polymorphisms in human leukocyte antigen genes and other immune response genes and possibly by exposomic factors, such as gut microbiome and enteral virus infections.^26^ These risk modifying factors can be shared between several IMDs. Thus, if HEDIMED project can identify exposomic factors common between different IMDs, its results can be generalized widely in the field studying the etiology of IMDs and other noncommunicable diseases.

## Project description

### Specific aims

The main goal of HEDIMED is to identify the complex interactions between external and internal exposomes and the key molecules and pathways that are critical for the development of T1D, CD, allergy, and asthma. HEDIMED fills critical knowledge gaps that have hindered the identification of exposomic determinants of IMDs and thereby facilitates the development of prevention and better treatments and diagnostics for IMDs.

This goal will be achieved by carrying out research to address the following specific objectives:

-  To identify and characterize the disease-specific and shared exposomic determinants of these IMDs and the biological pathways and mechanisms mediating their effects.
-  To develop new technologies for the characterization of the exposome and its role in IMDs.
-  To develop system dynamics modeling methods for simulating the effects of the exposome on the risk of IMDs and to create prediction models to estimate health economic savings of successful interventions.
- To carry out clinical intervention studies to evaluate whether the risk of IMDs can be reduced by targeted modulation of specific exposomic determinants.
-  To build a toolbox that allows various stakeholders to access the latest results and solutions to facilitate the development of novel diagnostics, therapeutic and prophylactic approaches for IMDs and to evaluate the public health consequences and costs.
- To communicate widely with various stakeholders and disseminate the key findings in an effective way.

### Who is in the study?

**Figure 3.**
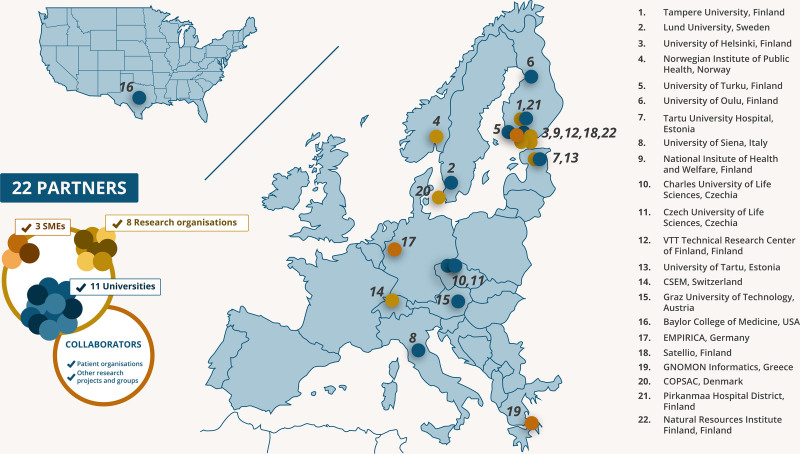
HEDIMED partners on the map.

HEDIMED brings together 22 partners from 12 different countries, including academic and public research organization and small- and medium-sized enterprises (SMEs), whose expertise covers medicine, clinical trials, epidemiology, public health science, nutritional science, microbiology, genetics, cell and molecular biology, chemistry and biochemistry, toxicology, soil ecology, immunoecology, geographical computer science, statistics, advanced modeling, and technical science. In addition to these scientific disciplines, there are actors who bring their expertise in communication and dissemination of research results and their exploitation to the HEDIMED (Figure 3).

## Clinical cohorts participating in HEDIMED

**Figure 4.**
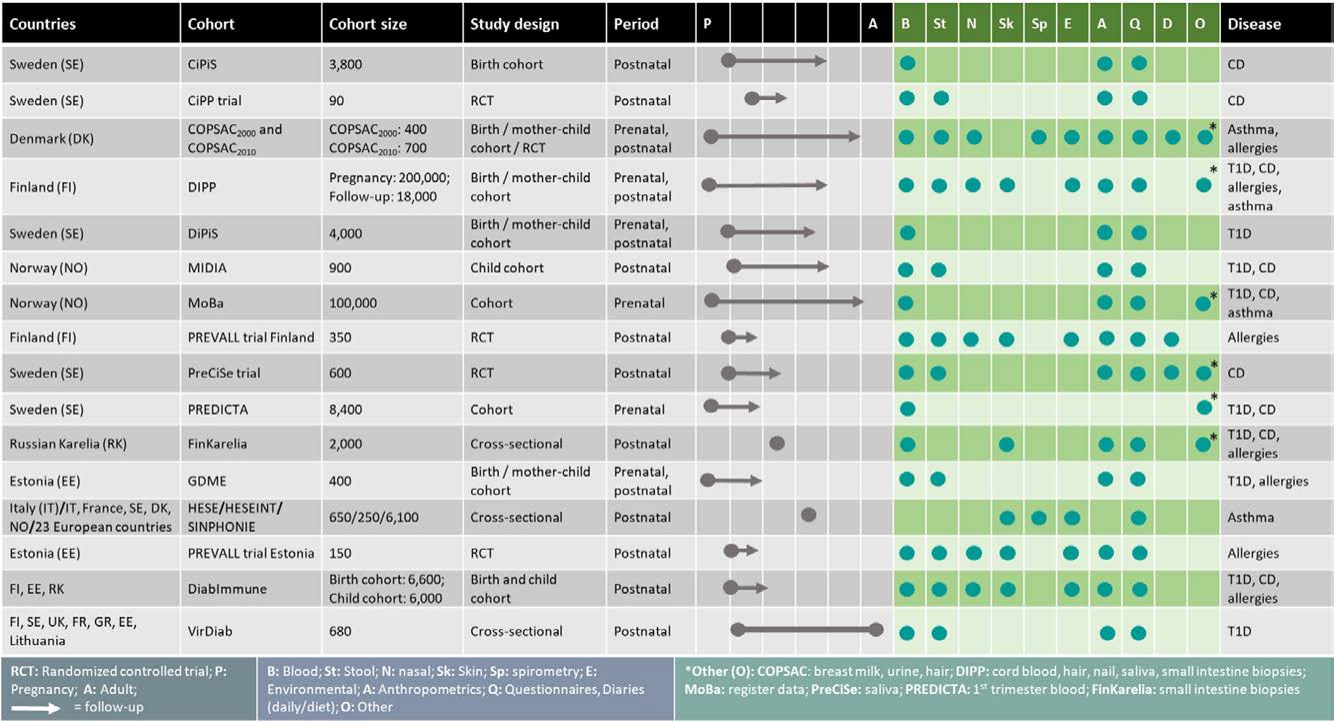
Overview of the key characteristics of cohorts used in HEDIMED.

The unique birth and cross-sectional cohorts form the basis of the research carried out in HEDIMED (Figure 4). These clinical cohorts provide data and biological samples from pregnant women and/or their offspring, prospectively followed from birth. In addition, HEDIMED includes clinical trials testing the effect of exposomic exposures on the immune system and the risk of IMDs. The risk of many IMDs is modulated by gender in a disease-specific manner,^33^ and the HEDIMED cohorts also allow the identification of these gender-specific exposomic determinants. These cohorts have been recruited in different populations but are based on similar study designs, which makes it possible to combine their data. Part of the analyzes will be carried out using cohort design, while some other analyzes will be carried out using nested-case control design.

**Figure 5.**
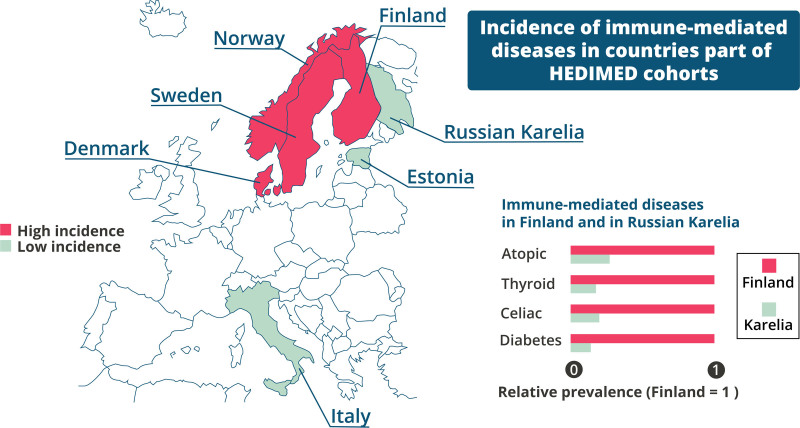
Incidence of IMDs in countries of HEDIMED cohorts. The bar chart in the bottom right corner highlights the difference seen in the relative prevalence of IMDs between Finland and Russian Karelia, where the populations share similar ancestry.

One of the virtues of HEDIMED is that these study populations represent countries with either high (Finland, Sweden, Norway, and Denmark) or lower (Italy, Estonia, and Karelian Republic of Russia) incidence of IMDs (Figure 5) creating an excellent opportunity to compare the differences in the exposome and its effects in the development of IMDs in these differing regions.^28,34^

## Approach

**Figure 6.**
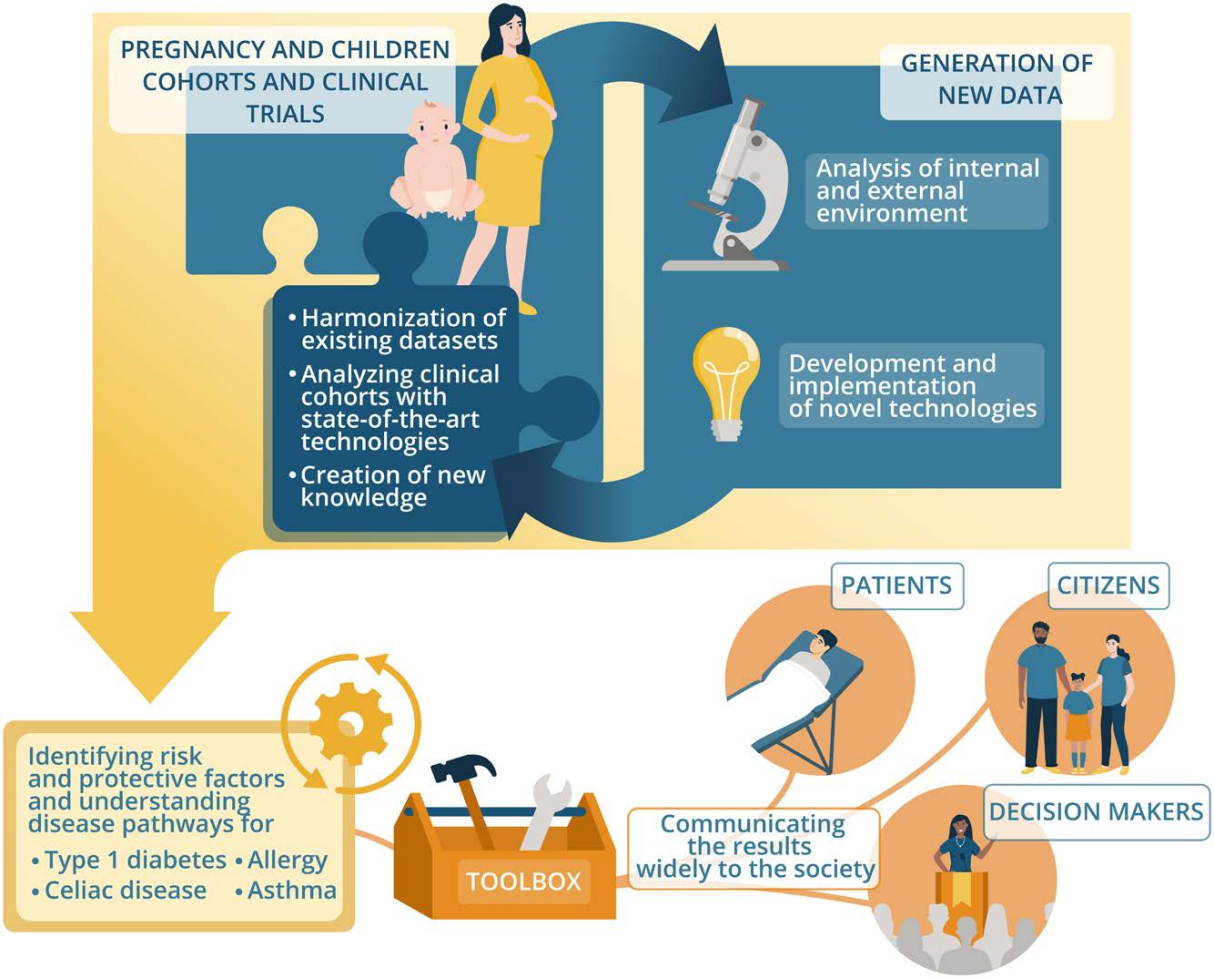
Overall design of the HEDIMED. Samples from 17 prospective cohorts and intervention trials will be analyzed for internal and external exposomic factors. The generated and existing data will be used to create a toolbox on the effects of the exposome on IMDs. The toolbox can be used by the public, decision-makers, patient organizations, and other stakeholders.

The methodological approach of HEDIMED can be roughly divided into five parts. First, HEDIMED brings together large data and sample sets from a number of pregnancy and child cohorts and clinical trials covering the IMDs of interest (T1D, CD, allergy, and asthma), the variables of which will be harmonized to allow integrated cross-cohort data analysis. HEDIMED will use the already existing data from these cohorts and complement it with new data created in the project. Second, HEDIMED will use state-of-the-art omics and other techniques to study the effects of both internal and external exposomic factors using the observational follow-up cohorts and early age trials with exposomic interventions. Health-related registry data will also be incorporated into the cohort studies. Third, the project will develop new intelligent sensors and analytical platforms to detect exposomic determinants (toxins and pollutants, environmental microbiome, infections, etc.) and cell and organoid models to study the mechanisms of observed associations. The fourth part includes activities devoted to computational data analysis and toolbox development: the project data combined with preexisting data will allow HEDIMED to build an exposome toolbox with data processing functions. Lastly, the toolbox with its various functionalities will enable the project know-how to be effectively disseminated to policymakers, the science community, regulatory authorities, patients, patient organizations, and industry. The toolbox can also model the medical and socioeconomic effects of exposomic determinants of IMDs and can predict the savings that the elimination of risk exposomic factors would generate to society. Schematic presentation of the overall project design is illustrated in Figure 6.

## Focus areas and methods

### Identification of study endpoints and creation of harmonized data and sample matrix in all clinical cohorts

One of the most important tasks is harmonizing the existing data and sample resources. Depending on the cohort, the samples and data range from the early 1990s´ to the present day. Data and samples collected in different cohorts, in different countries and during different decades need harmonization and new samples and data collected during HEDIMED will be added to this resource. An important harmonization effort is the definition of study endpoints in all cohorts. Background data will also be compared between the cohorts to enable the use of other variables in joint analyses (e.g., acute and chronic diseases, diet, infections, vaccinations, medications, and noncommunicable diseases in relatives).

### Measure the external exposome

To see which factors from the specific external environment impact the development of IMDs, HEDIMED aims to characterize acute and chronic infections encountered during pregnancy and in early childhood and the complete microbiome (bacteriome, virome, and parasitome) of the child and pregnant mothers. These will be examined using both metagenomic and targeted technologies, and novel multiplexing technologies will also be developed for these analyses. Other important research targets include immune system response to the commensal microflora in pregnant women and babies and in utero and postnatal exposure to selected environmental toxicants, such as persistent organic pollutants and perfluoroalkyl chemicals, which have been associated with immune-related outcomes^35–38^ and changes in commensal microbiota.^2^ One important factor that may influence disease outcome is the diet of pregnant mothers and babies. To this end, HEDIMED will characterize the study subjects’ exposure to various dietary compounds, such as breastmilk, fish-derived n-3 fatty acids, and cereals/gluten during in utero period and postnatally, using questionnaire data and dietary biomarkers, such as gliadin peptides. In addition, the microbiome of selected baby food items will be characterized.

One of the objectives of HEDIMED is to develop novel tools and affordable and scalable technologies to gain information about both past and acute exposures and interactions between the exposome and the host immune system. These technologies include arrays that can simultaneously detect a high number of target molecules and new volatilomics^39^ and metatranscriptomics technologies. The aim of these two latter studies is to understand how the bacterial species detected in, for example, stool or dust samples by metagenomic sequencing correlate with volatile compounds detected in these same samples and whether these volatile compounds can be used to characterize the metabolic activity of the microbiome and its correlations with the risk of IMDs. These technologies will facilitate the exposomic research in HEDIMED and more widely in the whole science community.^40^

HEDIMED has a unique possibility to characterize in detail general external exposomic determinants, such as the nature of the environment where the child has lived during the in utero period and during the first year of life. The land use around the home can be categorized into agricultural, rural, urban, and other types of environments using Global Positioning System coordinates and satellite data-based Corine land cover database.^41–44^ HEDIMED will use earth observation data and derived land cover and atmospheric variables, such as air quality and pollution, and combine it with other data, like season of birth, climate, and snow cover, to study these disease associations. More detailed analyses on the child’s living environment will be performed by characterizing the microbiota of the child’s home indoor and outdoor environment by taking, for example, floor mat samples^45^ indoors and yard soil samples outdoors.^46^

### Measure the internal exposome

Differences in the maturation of the immune system have been observed already in the analyses of umbilical cord blood transcriptomes of children born in regions with contrasting standards of living and hygiene and with differences in the prevalence of IMDs.^47^ This suggests that their immune system is differently programmed already during fetal life. HEDIMED will carry out extensive profiling of child´s internal environment during early life, using advanced omics, modeling, and data mining approaches to identify interactions between external exposures and the internal environment to look for phenotypic changes that are associated with IMDs. Profiling of child´s internal environment is based on blood proteomics and transcriptomics, cytokine profiling, and stool metabolomics. Transcriptomics will be utilized to analyze children’s responses to different exposomic factors and interventions to find out the activation of potentially diseases leading/protecting pathways.^48,49^ Other methods include epigenetics and characterization of circulating exosomes and small single-stranded non-coding RNA molecules. Also, the functions of innate immunity will be investigated by setting up short-term peripheral blood cell cultures using child’s blood samples, which in turn, will be stimulated by toll-like receptor agonists to analyze cytokine and gene expression responses. The observed properties of internal environment will be correlated to those of the external environment and the genetic polymorphisms and epigenetic status of each child.

### Evaluation of causality using clinical trials and experimental models

Clinical randomized intervention trials offer an advantage to study the mechanisms mediating the effect of the external exposome on the risk of IMDs in a standardized way. Therefore, they are considered as the strongest approaches to evaluate causality between exposomic determinants and IMDs.^50^ HEDIMED includes five intervention trials (Figure 4). In the PreCiSe trial (ClinicalTrials.gov Identifier: NCT03562221), the hypothesis is that a strict gluten-free diet during the first 3 years of life with a slow introduction of gluten will induce tolerance to gluten. Additionally, Prevention of Celiac Disease in Skåne and another Swedish trial celiac disease prevention with probiotics (ClinicalTrials.gov Identifier: NCT03176095) tested whether a daily delivery of two probiotics suppresses an inflammatory response to gluten.^51^ In the Danish Copenhagen Prospective Studies on Asthma in childhood study’s cohort, two randomized controlled trials were performed with the aim of preventing asthma (primary outcome measure) and allergic sensitization (one of the secondary outcome measures) in childhood. In a factorial design, pregnant mothers were given fish oil supplementation (n-3 fatty acids, ClinicalTrials.gov Identifier: NCT00798226) or matching placebo, and vitamin D3 (NCT00856947) or matching placebo from week 24 of pregnancy. Both of these pregnancy supplements showed protective effects on asthma development in the offspring.^52–55^ In the aforementioned trials, the interventions and sample collection have already been completed and the results partly published, but the biodiversity intervention and atopic sensitization trial (ClinicalTrials.gov Identifier: NCT03872219) is ongoing in Finland and in Estonia. In this trial, children will be exposed to environmental microbial biodiversity through nature-derived soil-based material from the forest during the first year of life in two countries with different environments.^56^ In addition to clinical trials, cell and organ culture models will be used to experimentally characterize those biological pathways that are activated by disease-associated exposomic determinants. This further helps to identify causal relationships as these experimental signatures can be correlated to those seen in vivo in clinical trials.

## Exposome data analysis and identification of disease associations

The complexity of the exposome (internal and external exposomes with numerous interacting variables) combined with the complexity of the human body and the development of IMDs, brings challenges to the statistical analysis of impact measurements. To this end and in contrast to most of the earlier exposome studies, HEDIMED will use a global exploratory approach to model the relations between different associations and identify shared or disease-specific biological pathways that most strongly predict the development of IMDs. In addition, the individual exposures that have been associated with the previously found outcomes (e.g., respiratory infections, enterovirus infections, rhinovirus infections, phospholipids, omega-3 fatty acids, gluten intake, breastfeeding, gut dysbiosis, exposure to high environmental microbial diversity, maternal virus antibodies, etc.)^24,57–63^ can be put into the wider context by analyzing them in the same model. Open-access data from other large cohorts and registry data will also be used as additional data sources. HEDIMED will use advanced data analytical tools and machine learning to model the complex multiexposure data and its associations with health outcomes and develop new tools for data fusion, novel data analysis, and data visualization.

## HEDIMED toolbox and data management

To give an overview of the exposome effects on IMDs, HEDIMED will develop a decision support software tool, referred to as the HEDIMED toolbox. The toolbox will have project-internal functions that support the researchers in their work and public functions to allow citizens and decision-makers utilize the public information. For example, it will include functions to estimate the societal and economic impacts of IMDs and to predict the impact of interventions. The toolbox will combine the data generated in HEDIMED and the data integrated from health registries to develop both population level and risk-group specific estimations. It will be based on the HEDIMED project internal research platform, which combines access to several datasets and tools for the analysis of the combined effects of several exposomic determinants on the risk of IMDs. The HEDIMED research platform will utilize the concept of data virtualization to provide access to up-to-date data sources from one place, and it allows researchers to write scripts for data analysis, modeling, visualization, and fetches the data from the cohort datasets as needed (without copying all data to one place). Public users can use the selected tools and data on a web server and explore the main results there. HEDIMED commits to the FAIR (i.e. data which meet principles of findability, accessibility, interoperability and reusability) data principles and the data and tools on the web server will be periodically updated.

### Ethical and data protection procedures

In HEDIMED, vast amounts of data from individuals from large human cohorts and clinical trials and their environment exist, are collected, used, and combined. This requires the ethical and data protection aspects to have a high priority to ensure that research is performed in a responsible way and all EU regulations, for example, General Data Protection Regulations (GDPRs), are followed in the sample and data analyses. In HEDIMED, the Ethical and Data Protection Committee, including representatives from each clinical cohort, was formed at the start of the project. Emphasis will be paid on the protection of personal identity of the study subjects using procedures legally accepted by EU member states. The aim is to create an anonymized database within project internal toolbox to allow flexible analyzes of the data for consortium members.

## Strengths and limitations

HEDIMED has multiple important strengths compared with previous studies allowing the identification of the exposomic determinants triggering the initiation and progression of IMDs. One important asset is the multidisciplinary consortium that combines foremost medical expertise with ecology, cutting-edge omics and other analysis methods, advanced know-how of intelligent sensors and world-class knowledge of data analysis and data mining tools. This assembly of leading-edge scientists in combination with strong support from the exploitation experts in the consortium, maximize the societal impacts of HEDIMED. This cornerstone of scientific excellence comprises of unique clinical cohorts and intervention studies that make it possible to identify prenatal and postnatal exposures in children who have been followed from fetal period until childhood and adolescence in countries with varying incidence of IMDs. One remarkable benefit is that, in addition to disease-specific exposomic determinants, HEDIMED will search for exposomic determinants that are common for several IMDs. The novel technological tools and solutions developed in HEDIMED, such as health-promoting exposomic interventions, provide foundations for developments that aim at healthier modern life.

Despite its obvious strengths, HEDIMED also has limitations. HEDIMED cohorts are constituted mainly of participants with Caucasian ancestry and therefore the associations found during the project should be verified in other populations as well. Another limitation is the complexity of the existing data, which needs to be harmonized between different cohorts. The same concerns samples and sampling. The samples in different cohorts have not necessarily been collected in exactly the same way, and therefore, extensive actions will be taken to harmonize the data, analytical platforms and the disease criteria used across the cohorts.

## Main challenges

One of the possible obstacles is legislation and regulation that concerns data privacy needed to connect different health data from cohorts and registry data outside cohorts. GDPR came into force in June 2018, and the practices and policies for its execution are still maturating in the member states. This may cause hindrances as practices in different countries may differ. Also, laws concerning data privacy, especially sensitive health data as used in HEDIMED, can vary between countries. The second challenging aspect is formed by data linkages between national registries and HEDIMED cohorts, which need close interaction with legislation and regulatory authorities to overcome any potential barriers. A long-term challenge with great impact is how the research findings and developed solutions can be effectively translated into new practices, guidelines, and recommendations. This needs an active and productive interaction between the HEDIMED consortium and different stakeholders including patient organizations, policy makers, lawmakers, local, regional, and regulatory authorities, and upper-level global organizations such as World Health Organization (WHO).

## Collaboration

One of the main aims of HEDIMED is to utilize and create collaboration networks that amplifies the scientific and societal impacts of the project. HEDIMED brings the wide collaboration networks of its partners available for the whole consortium. HEDIMED pays particular emphasis on collaboration with various stakeholders to increase the societal impact of the project including interactions with policy makers, patient organizations, municipal and national authorities and industry, and funding agencies that are operating in the field of IMDs. HEDIMED has an external advisory board including experts from the field of IMDs, genetics, microbiome, and epidemiology, which further facilitates the interactions with the science community. Most importantly, the European Human Exposome Network (EHEN) creates a huge and unique collaboration environment in this field in the EU and is an important cornerstone for and asset of HEDIMED. This network enables the use of resources and scientific expertise in studies aiming at the same goal—understanding the role of exposome in the pathogenesis of noncommunicable diseases.

## Conclusions

HEDIMED is the biggest endeavor focusing on the exposomic determinants of common IMDs. It utilizes a multidisciplinary research approach and combines samples and data from large clinical cohort studies. This combined sample and data matrix and state-of-the-art technologies provide an unprecedented opportunity to characterize relationships between exposomic determinants and IMDs and to recognize key biomarker molecules and pathways. More importantly, the sophisticated data analysis tools can, instead of identifying one determinant-one disease associations, recognize shared exposomic factors affecting several IMDs including type 1 diabetes, celiac disease, asthma, and allergy. The important outcome of the project will be the HEDIMED toolbox, which citizens, patients and patient organizations, policy makers and other authorities, and industry can utilize to develop actions aiming at healthier environment and interventions that can reduce the risk of IMDs. Altogether, HEDIMED represents a unique opportunity to generate critical new information and impact that can eventually lead to the development of prevention and better treatments for IMDs.

## Conflicts of interest statement

The authors declare that they have no conflicts of interest with regard to the content of this report.

This project has received funding from the European Union’s Horizon 2020 Research and Innovation Programme under Grant Agreement No: 874864.

## ACKNOWLEDGMENTS

HEDIMED is based on the common effort of all partners of the project including hundreds of investigators and other research personnel. Their contribution has been crucial in the design of the study and the background resources they have brought to the project is unique and highly prestigious. The clinical cohorts are based on long-term commitment of several partners to organize clinical studies and participation of hundreds of thousands of families in these studies. We also appreciate the assistance from the members of the wider European Human Exposome Network.

HEDIMED investigator group (P = partner): P1 Tampere University, Faculty of Medicine and Health Technology: Eurén Anna, Hyöty Heikki, Kurppa Kalle, Laiho Jutta, Laitinen Olli, Lehtonen Jussi, Lindfors Katri, Lönnrot Maria, Malkamäki Johannes, Numminen Henna, Nurminen Noora, Nykter Matti, Oikarinen Sami, Puustinen Leena, Saarinen Niila, Sioofy-Khojine Amirbabak, and Viiri Keijo; P2 Lund University, Department of Clinical Sciences: Agardh Daniel, Aronsson Carin Andrén, and Lundgren Markus; P3 University of Helsinki, Faculty of Biological and Environmental Sciences: Mäkelä Iida, Romantschuk Martin, and Soininen Laura; P4 The Norwegian Institute of Public Health: Nicolai A. Lund-Blix, Magnus Maria, Rantala Aino-Kaisa, Stene Lars, Størdal Ketil, and Tapia German; P5 University of Turku, Turku Bioscience Centre (University of Turku and Åbo Akademi University), Institute of Biomedicine, Department of Paediatrics: Elo Laura, Junttila Sini, Lahesmaa Riitta, Lempainen Johanna, Moulder Robert, Rasool Omid, Suomi Tomi, Toppari Jorma, and Ullah Ubaid; P6 University of Oulu, Department of Paediatrics: Veijola Riitta; P7 Tartu University Hospital: Peet Aleksandr, Simre Kärt, and Tillmann Vallo; P8 University of Siena, Department of Medicine, Surgery and Neuroscience: Bargagli Elena, Dotta Francesco, Nigi Laura, and Sebastiani Guido; P9 Finnish National Institute for Health and Welfare, Department of Health Security, Public Health Promotion Unit: Hakola Leena, Kiviranta Hannu, Rantakokko Panu, and Virtanen Suvi; P10 Charles University, 2nd Faculty of Medicine: Cinek Ondrej and Fronkova Eva; P11 Czech University of Life Sciences Prague, Department of Food Science: Havlik Jaroslav; P12 VTT Technical Research Centre of Finland: Barannik Emilia, Molinier Matthieu, Nevanen Tarja, Pajula Juha, Parmes Eija, Pärkkä Juha, Ranta Jukka, Rökman Jyri, Saviranta Petri, and Ylén Peter; P13 University of Tartu, Department of Immunology, Institute of Pharmacy: Aints Alar, Bärenson Anu, Kirss Anne, Laidmäe Ivo, Oras Astrid, Tagoma Aili, Uibo Raivo, and Vorobjova Tamara; P14 Swiss Center for Electronics and Microtechnology (CSEM): Burr Loïc, Cattaneo Stefano, Chai-Gao Hui, Cristofollini Peter, Generelli Silvia, Paoletti Samantha, and Ruth Edith; P15 Graz University of Technology, Institute of Environmental Biotechnology: Berg Gabriele and Wicaksono Wisnu; P16 Baylor College of Medicine, Department of Molecular Virology and Microbiology: Hoffman Kristi and Petrosino Joseph; P17 Empirica: Daniel Schmidtmann and Thiel Rainer; P18 Satellio: Salo Rosanna and Häme Lauri; P19 Gnomon: Berler Alexander, Karabatea Apostolia, and Papadopoulou Korina; P20 COPSAC: Bisgaard Hans, Bønnelykke Klaus, Brandt Sarah, Sevelsted Astrid, Stokholm Jakob, and Thorsen Jonathan; P21 Tampere University Hospital: Knip Mikael; and P22 Natural Resources Institute Finland, Horticulture Technologies: Roslund Marja and Sinkkonen Aki.

## References

[1] Maio S Baldacci S Carrozzi L. Respiratory symptoms/diseases prevalence is still increasing: a 25-yr population study. Respir Med. 2016;110:58–65. 26614595 10.1016/j.rmed.2015.11.006

[2] Roslund MI Rantala S Oikarinen S; ADELE team. Endocrine disruption and commensal bacteria alteration associated with gaseous and soil PAH contamination among daycare children. Environ Int. 2019;130:104894. 31220749 10.1016/j.envint.2019.06.004

[3] Lehel J Murphy S. Microplastics in the food chain: food safety and environmental aspects. Rev Environ Contam Toxicol. 2021;259:1–49. 34611754 10.1007/398_2021_77

[4] Parajuli A Grönroos M Kauppi S. The abundance of health-associated bacteria is altered in PAH polluted soils-implications for health in urban areas? PLoS One. 2017;12:e0187852. 29145477 10.1371/journal.pone.0187852 PMC5690629

[5] Mousavi SE Delgado-Saborit JM Adivi A Pauwels S Godderis L. Air pollution and endocrine disruptors induce human microbiome imbalances: a systematic review of recent evidence and possible biological mechanisms. Sci Total Environ. 2022;816:151654. 34785217 10.1016/j.scitotenv.2021.151654

[6] Flandroy L Poutahidis T Berg G. The impact of human activities and lifestyles on the interlinked microbiota and health of humans and of ecosystems. Sci Total Environ. 2018;627:1018–1038. 29426121 10.1016/j.scitotenv.2018.01.288

[7] Haahtela T von Hertzen L Anto JM. Helsinki by nature: the nature step to respiratory health. Clin Transl Allergy. 2019;9:57. 31695865 10.1186/s13601-019-0295-2 PMC6822361

[8] Haahtela T. A biodiversity hypothesis. Allergy. 2019;74:1445 1456. 30835837 10.1111/all.13763

[9] Renz H Allen KJ Sicherer SH. Food allergy. Nat Rev Dis Primers. 2018;4:17098. 29300005 10.1038/nrdp.2017.98

[10] Mowat AM. To respond or not to respond - a personal perspective of intestinal tolerance. Nat Rev Immunol. 2018;18:405–415. 29491358 10.1038/s41577-018-0002-x

[11] Herold KC Vignali DA Cooke A Bluestone JA. Type 1 diabetes: translating mechanistic observations into effective clinical outcomes. Nat Rev Immunol. 2013;13:243–256. 23524461 10.1038/nri3422 PMC4172461

[12] Fitzgibbon G Mills KHG. The microbiota and immune-mediated diseases: opportunities for therapeutic intervention. Eur J Immunol. 2020;50:326–337. 31991477 10.1002/eji.201948322

[13] Caminiti L Panasiti I Landi M. Allergen immunotherapy in atopic dermatitis: light and shadow in children. Pediatr Allergy Immunol. 2020;31(suppl 26):46–48. 10.1111/pai.13390 33236444

[14] Pajno GB Passanisi S Valenzise M Messina MF Lombardo F. The evolution of allergen-specific immunotherapy: the near and far future. Pediatr Allergy Immunol. 2020;31(suppl 26):11–13. 10.1111/pai.13351 33236435

[15] Pesce G Sesé L Calciano L. Foetal exposure to heavy metals and risk of atopic diseases in early childhood. Pediatr Allergy Immunol. 2021;32:242–250. 33091176 10.1111/pai.13397

[16] Narla S Silverberg JI. The role of environmental exposures in atopic dermatitis. Curr Allergy Asthma Rep. 2020;20:74. 33047271 10.1007/s11882-020-00971-z

[17] Peter I Maldonado-Contreras A Eisele C. A dietary intervention to improve the microbiome composition of pregnant women with Crohn’s disease and their offspring: the MELODY (Modulating Early Life Microbiome through Dietary Intervention in Pregnancy) trial design. Contemp Clin Trials Commun. 2020;18:100573. 32617430 10.1016/j.conctc.2020.100573 PMC7322804

[18] Wood TE Aksoy E Hachani A. From welfare to warfare: the arbitration of host-microbiota interplay by the type VI secretion system. Front Cell Infect Microbiol. 2020;10:587948. 33194832 10.3389/fcimb.2020.587948 PMC7604300

[19] Stewart CJ Ajami NJ O’Brien JL. Temporal development of the gut microbiome in early childhood from the TEDDY study. Nature. 2018;562:583–588. 30356187 10.1038/s41586-018-0617-x PMC6415775

[20] Lehtimäki J Karkman A Laatikainen T. Patterns in the skin microbiota differ in children and teenagers between rural and urban environments. Sci Rep. 2017;7:45651. 28361981 10.1038/srep45651 PMC5374497

[21] Adams K Weber KS Johnson SM. Exposome and immunity training: how pathogen exposure order influences innate immune cell lineage commitment and function. Int J Mol Sci. 2020;21:E8462. 33187101 10.3390/ijms21228462 PMC7697998

[22] Wild CP. Complementing the genome with an “exposome”: the outstanding challenge of environmental exposure measurement in molecular epidemiology. Cancer Epidemiol Biomarkers Prev. 2005;14:1847–1850. 16103423 10.1158/1055-9965.EPI-05-0456

[23] Cevhertas L Ogulur I Maurer DJ. Advances and recent developments in asthma in 2020. Allergy. 2020;75:3124–3146. 32997808 10.1111/all.14607

[24] Vatanen T Franzosa EA Schwager R. The human gut microbiome in early-onset type 1 diabetes from the TEDDY study. Nature. 2018;562:589–594. 30356183 10.1038/s41586-018-0620-2 PMC6296767

[25] Virtanen SM Takkinen HM Nwaru BI. Microbial exposure in infancy and subsequent appearance of type 1 diabetes mellitus-associated autoantibodies: a cohort study. JAMA Pediatr. 2014;168:755–763. 24957949 10.1001/jamapediatrics.2014.296

[26] Størdal K Kahrs C Tapia G Agardh D Kurppa K Stene LC. Review article: exposure to microbes and risk of coeliac disease. Aliment Pharmacol Ther. 2021;53:43–62. 33210316 10.1111/apt.16161

[27] Hyöty H. Viruses in type 1 diabetes. Pediatr Diabetes. 2016;17(suppl 22):56 64. 27411438 10.1111/pedi.12370

[28] Kondrashova A Seiskari T Ilonen J Knip M Hyöty H. The ‘hygiene hypothesis’ and the sharp gradient in the incidence of autoimmune and allergic diseases between Russian Karelia and Finland. APMIS. 2013;121:478–493. 23127244 10.1111/apm.12023

[29] Rewers M Hyöty H Lernmark Å; TEDDY Study Group. The Environmental Determinants of Diabetes in the Young (TEDDY) study: 2018 update. Curr Diab Rep. 2018;18:136. 30353256 10.1007/s11892-018-1113-2 PMC6415767

[30] Virtanen SM. Dietary factors in the development of type 1 diabetes. Pediatr Diabetes. 2016;17(suppl 22):49 55. 27411437 10.1111/pedi.12341

[31] Ojwang V Nwaru BI Takkinen HM. Early exposure to cats, dogs and farm animals and the risk of childhood asthma and allergy. Pediatr Allergy Immunol. 2020;31:265–272. 31829464 10.1111/pai.13186

[32] Stokholm J Blaser MJ Thorsen J. Maturation of the gut microbiome and risk of asthma in childhood. Nat Commun. 2018;9:141. 29321519 10.1038/s41467-017-02573-2 PMC5762761

[33] Korhonen L Oikarinen S Lehtonen J; DIABIMMUNE Study Group. Rhinoviruses in infancy and risk of immunoglobulin E sensitization. J Med Virol. 2019;91:1470–1478. 30866076 10.1002/jmv.25455

[34] Ruokolainen L Fyhrquist N Laatikainen T. Immune-microbiota interaction in Finnish and Russian Karelia young people with high and low allergy prevalence. Clin Exp Allergy. 2020;50:1148–1158. 32865840 10.1111/cea.13728 PMC7589450

[35] Cao J Xu X Hylkema MN. Early-life exposure to widespread environmental toxicants and health risk: a focus on the immune and respiratory systems. Ann Glob Health. 2016;82:119–131. 27325070 10.1016/j.aogh.2016.01.023

[36] Sinisalu L Sen P Salihović S. Early-life exposure to perfluorinated alkyl substances modulates lipid metabolism in progression to celiac disease. Environ Res. 2020;188:109864. 32846648 10.1016/j.envres.2020.109864

[37] Sargis RM Simmons RA. Environmental neglect: endocrine disruptors as underappreciated but potentially modifiable diabetes risk factors. Diabetologia. 2019;62:1811–1822. 31451869 10.1007/s00125-019-4940-z PMC7462102

[38] Crinnion WJ. Do environmental toxicants contribute to allergy and asthma? Altern Med Rev. 2012;17:6–18. 22502619

[39] Jokiniitty E Hokkinen L Kumpulainen P. Urine headspace analysis with field asymmetric ion mobility spectrometry for detection of chronic kidney disease. Biomark Med. 2020;14:629–638. 32613848 10.2217/bmm-2020-0085

[40] Vineis P Robinson O Chadeau-Hyam M Dehghan A Mudway I Dagnino S. What is new in the exposome? Environ Int. 2020;143:105887. 32619912 10.1016/j.envint.2020.105887

[41] Hanski I von Hertzen L Fyhrquist N. Environmental biodiversity, human microbiota, and allergy are interrelated. Proc Natl Acad Sci USA. 2012;109:8334–8339. 22566627 10.1073/pnas.1205624109 PMC3361383

[42] Parmes E Pesce G Sabel CE. Influence of residential land cover on childhood allergic and respiratory symptoms and diseases: evidence from 9 European cohorts. Environ Res. 2020;183:108953. 31818476 10.1016/j.envres.2019.108953

[43] Nurminen N Cerrone D Lehtonen J. Land cover of early-life environment modulates the risk of type 1 diabetes. Diabetes Care. 2021;44:1506–1514. 33952607 10.2337/dc20-1719 PMC8323192

[44] Vari HK Roslund MI Oikarinen S; ADELE research group. Associations between land cover categories, gaseous PAH levels in ambient air and endocrine signaling predicted from gut bacterial metagenome of the elderly. Chemosphere. 2021;265:128965. 33248729 10.1016/j.chemosphere.2020.128965

[45] Hui N Parajuli A Puhakka R. Temporal variation in indoor transfer of dirt-associated environmental bacteria in agricultural and urban areas. Environ Int. 2019;132:105069. 31400602 10.1016/j.envint.2019.105069

[46] Puhakka R Rantala O Roslund MI Rajaniemi J Laitinen OH Sinkkonen A; ADELE Research Group. Greening of daycare yards with biodiverse materials affords well-being, play and environmental relationships. Int J Environ Res Public Health. 2019;16:E2948. 31426345 10.3390/ijerph16162948 PMC6719197

[47] Kallionpää H Laajala E Öling V; DIABIMMUNE Study Group. Standard of hygiene and immune adaptation in newborn infants. Clin Immunol. 2014;155:136–147. 25245264 10.1016/j.clim.2014.09.009

[48] Lietzen N An LTT Jaakkola MK. Enterovirus-associated changes in blood transcriptomic profiles of children with genetic susceptibility to type 1 diabetes. Diabetologia. 2018;61:381–388. 29119244 10.1007/s00125-017-4460-7 PMC6448961

[49] Moulder R Lahesmaa R. Early signs of disease in type 1 diabetes. Pediatr Diabetes. 2016;17(suppl 22):43 48. 27411436 10.1111/pedi.12329

[50] Andrianou XD Pronk A Galea KS. Exposome-based public health interventions for infectious diseases in urban settings. Environ Int. 2021;146:106246. 33181410 10.1016/j.envint.2020.106246 PMC7834142

[51] Håkansson Å Andrén Aronsson C Brundin C Oscarsson E Molin G Agardh D. Effects of lactobacillus plantarum and lactobacillus paracasei on the peripheral immune response in children with celiac disease autoimmunity: a randomized, double-blind, placebo-controlled clinical trial. Nutrients. 2019;11:1925. 10.3390/nu11081925 PMC6723580 31426299

[52] Bisgaard H Stokholm J Chawes BL. Fish oil-derived fatty acids in pregnancy and wheeze and asthma in offspring. N Engl J Med. 2016;375:2530–2539. 28029926 10.1056/NEJMoa1503734

[53] Chawes BL Bønnelykke K Stokholm J. Effect of vitamin D3 supplementation during pregnancy on risk of persistent wheeze in the offspring: a randomized clinical trial. JAMA. 2016;315:353–361. 26813208 10.1001/jama.2015.18318

[54] Wolsk HM Chawes BL Litonjua AA. Prenatal vitamin D supplementation reduces risk of asthma/recurrent wheeze in early childhood: a combined analysis of two randomized controlled trials. PLoS One. 2017;12:e0186657. 29077711 10.1371/journal.pone.0186657 PMC5659607

[55] Brustad N Eliasen AU Stokholm J Bønnelykke K Bisgaard H Chawes BL. High-dose vitamin D supplementation during pregnancy and asthma in offspring at the age of 6 years. JAMA. 2019;321:1003–1005. 30860552 10.1001/jama.2019.0052 PMC6439670

[56] Nurminen N Lin J Grönroos M. Nature-derived microbiota exposure as a novel immunomodulatory approach. Future Microbiol. 2018;13:737–744. 29771153 10.2217/fmb-2017-0286

[57] Laitinen OH Honkanen H Pakkanen O. Coxsackievirus B1 is associated with induction of β-cell autoimmunity that portends type 1 diabetes. Diabetes. 2014;63:446–455. 23974921 10.2337/db13-0619

[58] Sioofy-Khojine AB Lehtonen J Nurminen N. Coxsackievirus B1 infections are associated with the initiation of insulin-driven autoimmunity that progresses to type 1 diabetes. Diabetologia. 2018;61:1193–1202. 29404673 10.1007/s00125-018-4561-y

[59] Feddema JJ Claassen E. Prevalence of viral respiratory infections amongst asthmatics: results of a meta-regression analysis. Respir Med. 2020;173:106020. 33190740 10.1016/j.rmed.2020.106020

[60] Read JF Bosco A. Decoding susceptibility to respiratory viral infections and asthma inception in children. Int J Mol Sci. 2020;21:E6372. 32887352 10.3390/ijms21176372 PMC7503410

[61] Khan MI Hariprasad G. Human secretary phospholipase A2 mutations and their clinical implications. J Inflamm Res. 2020;13:551 561. 32982370 10.2147/JIR.S269557 PMC7502393

[62] Dignass AU. Mechanisms and modulation of intestinal epithelial repair. Inflamm Bowel Dis. 2001;7:68–77. 11233665 10.1097/00054725-200102000-00014

[63] Olshan KL Leonard MM Serena G Zomorrodi AR Fasano A. Gut microbiota in celiac disease: microbes, metabolites, pathways and therapeutics. Expert Rev Clin Immunol. 2020;16:1075–1092. 33103934 10.1080/1744666X.2021.1840354 PMC7796936

